# Esophagus foreign body in the thyroid gland^☆^^☆☆^

**DOI:** 10.1016/j.bjorl.2017.08.008

**Published:** 2017-09-02

**Authors:** Sílvia Miguéis Picado Petrarolha, Rogério Aparecido Dedivitis, Fabíola Garcia Perruccio, Ingrid de Andrade Quirino

**Affiliations:** a Hospital Ana Costa, Departamento de Cirurgia de Cabeça e Pescoço, Santos, SP, Brazil; b Universidade de São Paulo (USP), Faculdade de Medicina, Departamento de Cirurgia de Cabeça e Pescoço, São Paulo, SP, Brazil; c Universidade Metropolitana de Santos (UNIMES), Santos, SP, Brazil

## Introduction

A migratory esophageal foreign body (FB) is uncommon and migration of an FB into the thyroid gland is rare.^1^, ^2^ To our knowledge, only 22 such cases have been reported in the English-language literature.^1^, ^3^ Due to their fine, linear and sharp structure, fish bones have a tendency to become lodged and penetrate the esophageal mucosa into the thyroid gland space because of the swallowing movement.^2^, ^3^ If left untreated, serious and potentially fatal complications can develop, such as peroesophagitis, periesophageal abscess, mediastinitis, aortoesophageal fistula, innominate esophageal fistula and carotid rupture.^1^, ^4^ Prompt diagnosis is essential in the management of a perforating FB.^1^

In this case report the patient had an abscess formation due to the presence of an FB in the level of the thyroid gland.

## Case report

**Figure 1:**
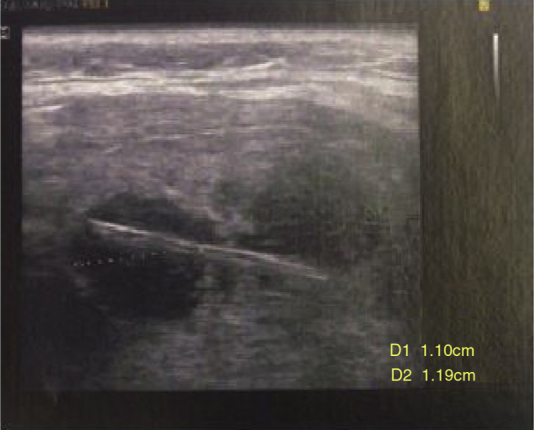
Hyperdense linear image inside the left thyroid lobe, with associated content and debris.

**Figure 2:**
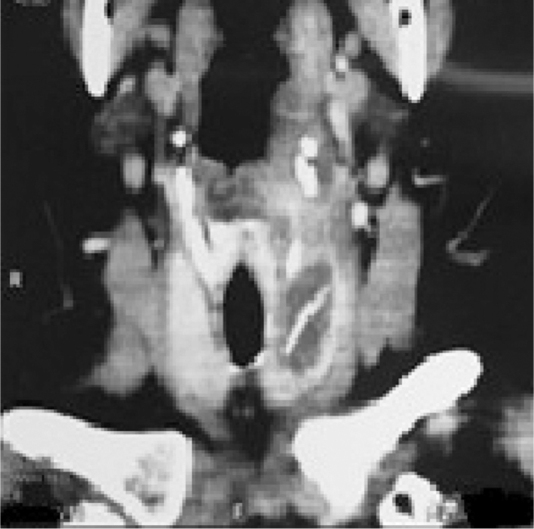
CT showed juxtaposed collection on the left thyroid lobe with a foreign body inside.

**Figure 3:**
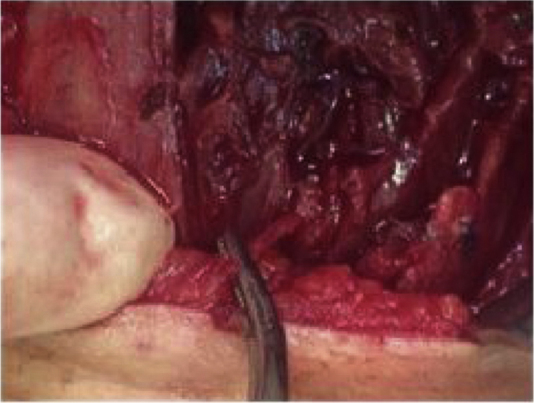
Foreign body adjacent to the trachea, lying 2 mm from the usual path of the ipsilateral recurrent laryngeal nerve.

**Figure 4:**
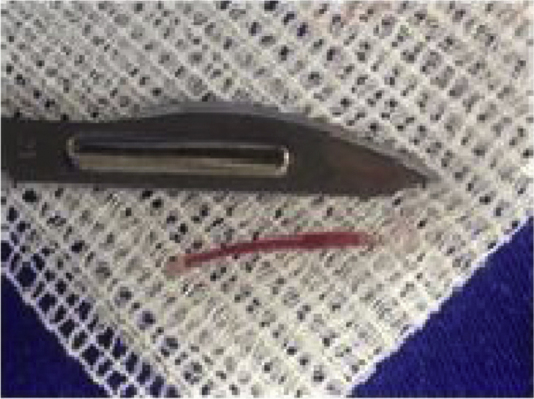
Fish bone.

A 67 year-old woman was admitted in the emergency room complaining of dysphagia and pain in the cervical region, especially in the left side of the neck. Symptoms had started 9 days before, with progression of the painful episodes. She reported an accidental ingestion of a fish bone in the period. There was no improvement with the use of anti-inflammatory or analgesic drugs. On physical examination, cervical bulging, discreet dyspnea and no fever were noted; lab exam showed leukocytosis. Cervical ultrasonography showed a hyperdense linear image inside the left thyroid lobe, with associated content and debris, and a perforation in the esophagus (Fig. 1). Cervical computed tomography (CT) revealed a juxtaposed collection in the left thyroid lobe with a foreign body inside (Fig. 2). Exploratory cervicotomy was performed in order to drain the abscess and remove the FB. It was possible to observe significant fibrosis and soft tissue edema. The foreign body was juxtaposed to the trachea, lying 2 mm from the usual path of the ipsilateral recurrent laryngeal nerve (Figure 3, Figure 4). The patient remained with a nasoenteral tube for 5 days and oral diet was given after that. Antibiotics were started and continued for 12 days. She was discharged on the 7th day without complaint and returned for follow up without symptoms.

## Discussion

When fish bones become lodged, this usually occurs in the palatine tonsil, the base of the tongue, vallecula, pyriform sinus and esophagus.^5^, ^6^ The location of a fish bone outside the pharynx and the subsequent formation of a thyroid abscess is extremely rare.^2^, ^5^ The longer the FB is impacted in the esophagus, the higher the risk of perforation, and for this reason prompt diagnosis is essential.^1^, ^2^

Plain radiography of the neck is frequently useful; however, images of the FB and thyroid cartilage sometimes overlap and this technique is thus not sensitive enough for consistent identification of fish bone.^6^, ^7^ CT scan offers better detection of thin, small, minimally calcified foreign bodies.^4^ It is also an essential preoperative investigation as it confirms that an esophageal foreign body has migrated.^4^ Some studies have been published on the usefulness of the CT for cases in which an impacted fish bone in the pharynx-esophagus is suspected.^1^, ^2^, ^3^ In our case, we identified the fish bone by using ultrasonography and confirmed its extension with the CT.

## Conclusion

Fish bone in the thyroid gland is rare and difficult to diagnose due to the lack of severe or characteristic symptoms.^1^, ^5^ However, it is very important to remove it in a timely manner. Surgical extirpation is usually performed, but thyroidectomy is not always necessary.^5^

## Conflicts of interest

The authors declare no conflicts of interest.
